# Purely ropivacaine-based TEA vs single TAP block in pain management after elective laparoscopic colon surgery within an upgraded institutional ERAS program

**DOI:** 10.1007/s00464-021-08647-z

**Published:** 2021-09-03

**Authors:** Vilma Bumblyte, Suvi K. Rasilainen, Anu Ehrlich, Tom Scheinin, Vesa K. Kontinen, Aino Sevon, Heikki Vääräniemi, Alexey A. Schramko

**Affiliations:** 1 Department of Anaesthesiology, Intensive Care and Pain Medicine, University of Helsinki and Helsinki University Hospital, Jorvi Hospital, P.O. Box 00029 HUS, Espoo, Finland; 2 Department of Gastrointestinal Surgery, University of Helsinki and Helsinki University Hospital, Jorvi Hospital, Espoo, Finland; 3 Department of Surgery and Department of Anaesthesiology, Central Finland Central Hospital, Jyvaskyla, Finland; 4 Medical Faculty, University of Helsinki, Helsinki, Finland

**Keywords:** Laparoscopic colon surgery, ERAS (enhanced recovery after surgery), Pain management, Transversus abdominis plane block, Thoracic epidural analgesia

## Abstract

**Background:**

The aim of this study was to compare thoracic epidural analgesia (TEA) with transversus abdominis plane (TAP) block in post-operative pain management after laparoscopic colon surgery.

**Methods:**

One hundred thirty-six patients undergoing laparoscopic colon resection randomly received either TEA or TAP with ropivacaine only. The primary endpoint was opioid requirement up to 48 h postoperatively. Intensity of pain, time to onset of bowel function, time to mobilization, postoperative complications, length of hospital stay, and patients’ satisfaction with pain management were also assessed.

**Results:**

We observed a significant decrease in opioid consumption on the day of surgery with TEA compared with TAP block (30 mg vs 14 mg, *p* < 0.001). On the first two postoperative days (POD), the balance shifted to opioid consumption being smaller in the TAP group: on POD 1 (15.2 mg vs 10.6 mg; p = 0.086) and on POD 2 (9.2 mg vs 4.6 mg; *p* = 0.021). There were no differences in postoperative nausea/vomiting or time to first postoperative bowel movement between the groups. No direct blockade-related complications were observed and the length of stay was similar between TEA and TAP groups.

**Conclusion:**

TEA is more efficient for acute postoperative pain than TAP block on day of surgery, but not on the first two PODs. No differences in pain management-related complications were detected.

**Supplementary Information:**

The online version contains supplementary material available at 10.1007/s00464-021-08647-z.

Effective pain management with minimal adverse effects is crucial for successful postoperative recovery. Recently, the standards of patient care after elective surgery worldwide have turned toward enhanced recovery after surgery (ERAS) pathways, which highlight the importance of effective but opioid-sparing methods in pain relief [1]. Epidural analgesia has been considered an essential component in ERAS due to its potential for diminishing the stress response, improving intestinal circulation, and thus decreasing intestinal paralysis [2–4]. It has been recognized as the most effective opioid-sparing method [5, 6]. This key role of epidural analgesia has been questioned in recent years, especially in laparoscopic surgery. While Jouve et al. [7] have demonstrated that epidural analgesia speeds up recovery and reduces opioid consumption and related adverse effects after open colon surgery, opposite results have been found in other concurrent studies [8].

In laparoscopic colon surgery, the benefit from epidural analgesia is even more unclear. A meta-analysis in 2013 concluded that epidural analgesia had some beneficial effects on pain relief and return of bowel function but no effect on length of stay (LOS) [9]. Furthermore, a major retrospective study of 4102 patients with laparoscopic colorectal resection showed epidural analgesia to be safe but to increase LOS, infections, and costs [10]. Our study group has previously reported benefits in pain relief but otherwise similar results for epidural analgesia compared to patient-controlled analgesia (PCA) in patients with laparoscopic sigmoid resection treated within an ERAS program [11]. This setting was further tested by Hübner et al. in 128 laparoscopic colorectal resection patients with a conclusion of increased adverse effects and no recommendation for routine use in laparoscopic colorectal surgery [12].

To search for new insights, many studies have been conducted on diverse analgesic techniques. In a meta-analysis, transversus abdominis plane (TAP) block was shown to reduce opioid consumption and to improve bowel function after laparoscopic colorectal surgery [13]. In another review, TAP block led to lower pain scores and to reduced opioid consumption after laparoscopic colorectal surgery, compared with no intervention, saline injections, or other techniques [14].

The most important patient-centered outcomes after colonic surgery are return to normal life or recovery without surgical complications or prolonged hospitalization, life quality, and, in the immediate postoperative period, adequate pain management with minimal adverse effects. When pain management methods, such as epidural analgesia and TAP block, are studied, it is pertinent to concentrate on outcomes related to analgesia. As it would be unethical to let any patient to suffer from intense pain without supplementary analgesics, opioid consumption is often used as a proxy for the adequacy of analgesia.

Thoracic epidural analgesia (TEA) is a technique associated with rare, but potentially serious, adverse effects. Therefore, to justify using it as a component of perioperative management of laparoscopic colon resection patients, it would have to be associated with significant benefit for pain control. In different types of acute postoperative pain, a decrease of 18–35% in opioid consumption has in most studies been considered to represent a minimal clinically important benefit [15]. Thus, we wanted to be able to detect a decrease of at least 20% in opioid consumption.

The aim of this study was to compare ropivacaine-based TEA with TAP block for postoperative pain management in patients undergoing elective laparoscopic colon surgery. As the adverse effects of pain management are related to size of opioid dose used [16, 17], opioid consumption was chosen as the primary outcome and a decrease of at least 20% in the epidural group compared with the TAP group was considered to be clinically significant.

## Materials and methods

### Study design

Written informed consent was obtained from all study patients. This trial was a prospective parallel group randomized superiority trial comparing TEA with TAP block in patients undergoing elective laparoscopic colon surgery, with a 1:1 allocation ratio. Patients were recruited from January 2018 until May 2019. The trial was registered prior to patients’ enrollment in EU Clinical Trials Register (EudraCT-nr: 2017-001714-29). The trial was performed in Helsinki University Hospital and Central Finland Central Hospital. The research plan was approved by the local ethics committee of Helsinki University Hospital and by the institutional review board at both Helsinki University Hospital and Central Finland Central Hospital. This manuscript adheres to the relevant CONSORT guidelines.

### Patients

All patients scheduled for elective laparoscopic colon resection were considered as candidates for inclusion. The exclusion criteria were severe renal insufficiency (GFR < 30 ml/min), severe hepatic insufficiency (TT ≤ 50%), severe COPD (FEV1 > 30%), metastatic malignancy, hematologic disease or a congenital clotting disorder, preoperative opioid use, age under 18 years, pregnancy or breast-feeding, hyper-reactivity toward ropivacaine, estimated risk for conversion to open surgery > 50%, some other contra-indication to epidural catheterization (e.g., patient’s wishes).

Recruitment of patients took place between January 10, 2018 and July 1, 2019.

### Randomization

If eligible, patients were given detailed written and verbal information on the trial. Based on this, informed written consent was obtained and patients randomly enrolled in blocks of 16 (right- and left-sided colectomies separately) for either TEA or TAP block. The randomization sequence was generated by a web-based service. The sequence was concealed in opaque numbered envelopes, which were opened in numerical order (sealed envelopes method). In view of ethical issues associated with an invasive procedure and the insertion of epidural catheters for patients in the TAP group, the study was not blinded to patients or to the treating physicians or nurses. Furthermore, if placebo epidurals had been used, this would have been revealed to patients and personnel in the recovery room due to lack of any local anesthetic effect (numbness) on the truncal skin. The patients’ data were coded in CRF; therefore, outcomes assessors were blinded to the interventions.

### Treatments and interventions

All patients were treated according to our ERAS program, optimized for elective colon surgery. Preoperative preparations were consistently performed for all enrolled patients, both at the preoperative polyclinic and at the admitting unit for elective surgery. Clear liquids were allowed up to 2 h and solids up to 6 h prior to induction of anesthesia. Two hours before anesthesia, carbohydrate drinks were given. For antibiotic prophylaxis, cefuroxime–metronidazole single dose was administered after anesthesia induction. For patients with hypersensitivity to cephalosporin, ciprofloxacin was used. If the surgery lasted longer than 3 h, another dose of cefuroxime was given. Prophylaxis against thromboembolism was accomplished with thigh-high compression stockings and enoxaparin starting 6 h after operation.

All patients received paracetamol 1 g orally or intravenously at the pre-surgery unit or before induction of anesthesia. For preoperative anxiety, 5–10 mg of temazepam was given orally on request 1 h before induction of anesthesia.

### Anesthesia protocol and pain management

For anesthesia induction, propofol 1–2.5 mg/kg was given. General anesthesia was maintained with an inhalation anesthetic (sevoflurane or desflurane in oxygen and air mixture 0.8–1.2 MAC) or intravenously administered propofol. During anesthesia, 0.05–0.1 mg iv of fentanyl boluses was administered as an analgesic. Rocuronium was used as a muscle relaxant 0.3–0.5 mg/kg during induction and 10–20 mg iv boluses for muscle relaxation maintenance. At the end of surgery, relaxation was reversed by administration of neostigmine–glycopyrronium (Glycostigmin®) 1.0 ml or sugammadex 1–4 mg/kg iv. All patients received 5 mg of dexamethasone at anesthesia induction for postoperative nausea/vomiting (PONV) prophylaxis. Postoperatively, PONV medication was given on request according to the hospital guidelines. Depending on the patient's co-morbidities, non-invasive or invasive blood pressure monitoring was performed, aiming at normotension (mean arterial pressure 50–70 mmHg). If necessary, anesthesia-induced hypotension was treated with norepinephrine infusion.

As fluid therapy, Ringer’s acetate solution 2–4 ml/kg/h infusion was administrated intravenously during surgery.

Intraoperative bleeding of less than 500 ml was not replaced; for bleeding of 500–1000 ml, Ringer's acetate solution 1:1 was administered; for bleeding higher than 1000 ml, colloid solution (4% Albumin) was considered in addition to Ringer’s acetate solution. Anemia (hemoglobin below 85 g/l) was corrected by administering a red blood cell transfusion. After surgery, Ringer's solution was infused slowly (30–50 ml/h).

Patients were weaned from the ventilator and extubated in the operating room. Nasogastric tubes were applied only at surgeons’ request and were removed before extubation. Urine catheters were removed on the first or second postoperative day (POD). Drains were not routinely used.

For postoperative analgesia, all patients received paracetamol 0.5–1 g × 3 po/iv, starting immediately after surgery, and ibuprofen 400–800 mg × 3 po from the 1POD. NSAIDs have not been administered in cases of renal injury (postoperative oliguria or creatinine raising on the 1POD more than 50% from preoperative level). Opioids were given only on request (pain numerical rating scale (pain NRS) 0–10, for pain NRS > 3 at rest or for pain NRS > 5 on exercise): oxycodone 0.05 mg/kg iv (only in the recovery room), 0.07–0.1 mg/kg im or 0.15–0.2 mg/kg po was used.

In the recovery room, fluids were given orally 1–1.5 h after surgery (in the absence of nausea). The patients were mobilized in the recovery room 1.5–2 h after surgery.

### Application of epidural catheters

For patients in the TEA group, epidural catheters were placed preoperatively. Immediately after this, lidocaine and epinephrine (20 mg/ml + 5 µg/ml) solution (3 ml) was administered via epidural catheter. After 5 to 10 min, the increase in heart rate and numbness of the lower limbs were assessed in order to detect erroneous intravenous or intrathecal catheter placement.

Ropivacaine 2 mg/ml infusion was started at 2–4 ml/h at anesthesia induction. At the end of surgery, the infusion rate was increased to 6–8 ml/h. Postoperatively, the infusion rate was adjusted, according to pain relief response and side effects, to 2–12 ml/h, continuing up to 48 h postoperatively.

### TAP block

All surgeries were performed by specialized colorectal surgeons. Bilateral TAP block was administered intraoperatively at laparoscopy, directly after insufflation and insertion of the first trocar and camera. Under good visibility, an 18-gauge needle was inserted through skin until two ‘pops’ had occurred, indicating entrance into the TAP plane (intramuscular space between internal oblique fascia and transversus abdominis muscle). The ropivacaine was infused as a bolus of 40 ml, 5 mg/ml, or 3.75 mg/ml (20 ml per side). Before incision, surgical wounds were infiltrated with ropivacaine solution 5 mg/ml, 15 ml. The maximum single dose of ropivacaine was 4 mg/kg.

### Outcomes and measures

The primary outcome measure was opioid consumption during the first 48 h postoperatively. Secondary outcome measures included pain intensity on a numerical rating scale (pain NRS, 0–10). In the recovery room, pain intensity was measured 30 min after arrival and then every hour until the patient was discharged to the ward. In the ward, the pain NRS score was measured for each consecutive 6-h block. Means of the first and second 6-h blocks and third and fourth blocks were calculated and used for further analysis. Additionally, the maximal daily pain NRS scores were collected. At the time of discharge, patients evaluated their satisfaction with pain management as a whole (scale 0–3: 0 poor; 3 excellent).

At 2 to 4 weeks postoperatively, all patients were contacted either by phone or at the outpatient clinic during a follow-up visit and asked to complete a form with questions evaluating pain sensations, pain intensity, need for pain medication, performance of daily activities, and any limitations of these due to postoperative abdominal pain.

Other secondary outcome measures included first postoperative bowel movement (gas or solid excrement) post-operatively, intraoperative fluid balance, postoperative complications (recorded by Clavien–Dindo classification), readmission rate, and LOS. Furthermore, the power of epidural analgesia was compared in pre-hoc-designed subgroups for right and left colectomies.

### Sample size

When this study was planned, there were no applicable direct comparison studies between epidural analgesia and TAP block in laparoscopic colon surgery available as a basis for the sample size calculation. We wanted to be able to detect a 20% decrease in opioid consumption. In a recent study, the mean PCA morphine consumption after laparoscopic colon resection was 31.3 mg (standard error (SE) 3.8) in the TAP group. Taking into account the transformation from SE to SD, conversion from morphine to oxycodone and the move from iv to im/po administration, an SD of 18.6 was chosen for the power calculation. With *α* = 0.05 and 1 − *β* = 0.9, 69 patients per group (total 138) would be needed to show this difference. Taking into account the potential dropout rate of 15%, the sample size was set at 160.

### Statistical analysis

Statistical analyses were performed using IBM SPSS Statistics 22 software. Statistical significance was set at a two-sided *α* of 0.05. The distribution of the patient population was tested with the Kolmogorov–Smirnov test. Accordingly, both non-parametric tests (Mann–Whitney *U* test, comparing medians across groups) and one-way ANOVA were used to analyze continuous variables. Categorical variables were analyzed by Pearson’s chi-squared test and Fisher’s exact test.

## Results

**Fig. 1:**
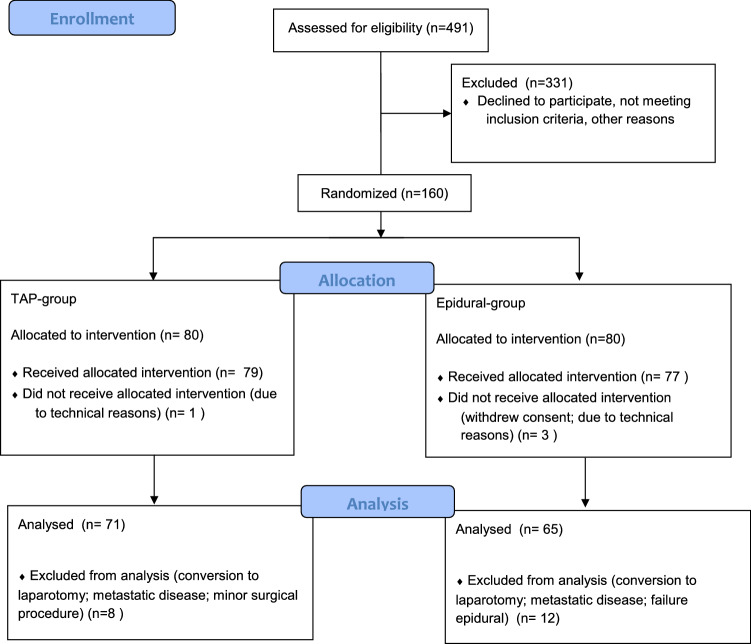
Study flow diagram

One hundred and sixty patients who gave written informed consent were enrolled and randomly assigned to TAP block or TEA; 24 patients dropped out of the study after enrollment for various reasons (Fig. 1); 136 patients were included in the statistical analysis, 71 for TAP block and 65 for TEA. There were no significant differences between groups in baseline characteristics (Table 1).

**Table 1:** Patient characteristics

	TAP (*n* = 71)	TEA (*n* = 65)	*p*
Age			
Mean	68.47	67.88	
Median (range)	70 (33–87)	69 (45–91)	0,7
BMI^a^			
Mean	26.11	26.614	0,8ͨ
Median	26.00	25.8	
Gender (%)			0,3
Male	28 (39.4)	32 (50)	
Female	41	32	
ASA^b^ (%)			0,9
I	3 (4.2)	4 (6,2)	
II	41 (57.7)	36 (55.4)	
III	26 (36.6)	24 (36.9)	
IV	1 (1.4)	1 (1.5)	
Comorbidity (%)			0,7
Hypertension	39 (54.9)	34 (52.3)	
Asthma/COPD *c*	14 (19.7)	9 (13.8)	
DM^d^	13 (18.3)	13 (20.0)	
Other	4 (6.2)	5 (7.0)	
Smoker (%)	9 (12.7)	5 (7.7)	0,3
Malignant (%)	54 (76)	46 (70.8)	0,5
Colectomy (%)			0,2
Right	38 (53.5)	28 (39.4)	
Left	33 (46.5)	34 (60.6)	

^a^*BMI* body mass index

^b^*ASA* physical status classification system

^c^*COPD* chronic obstructive pulmonary disease

^d^*DM* diabetes mellitus

### Opioid consumption within 48 h postoperatively and pain NRS scores

Patients in the TEA group needed significantly lower amounts of opioids on the day of surgery than those in the TAP group, *p* < 0.001 (Table 2). On POD 1, the median opioid demand was similar in both groups, *p* = 0.09. In contrast, on POD 2, the TEA group needed significantly more than the TAP group, *p* = 0.015. In line with our hypothesis, the overall opioid consumption during the first 48 h postoperatively was reduced with TEA by 20%, compared with TAP block (median 29 mg vs 40 mg, respectively) (Table 2).

**Table 2:** Opioid requirement 48 h after surgery

	TAP *n* = 71	TEA *n* = 65	*p*
0 POD	28 (10–50)	10 (0–20)	< 0.0001
1 POD	10 (0–15)	10 (0–25)	0.09
2 POD	0 (0–5)	5 (0–20)	0.02
Cumulative	40 (12–60)	29 (10–52)	0.3

Values are in morphine equivalents, oral doses in milligrams

Values are median (quartile)

*POD* postoperative day

**Fig. 2:**
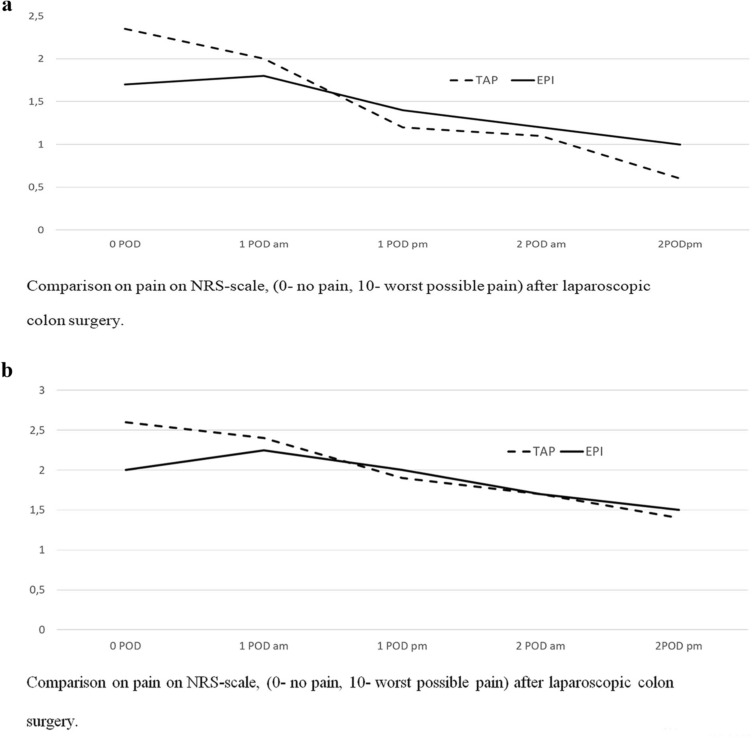
Comparison of pain (pain NRS: 0 no pain; 10 worst possible pain) after laparoscopic colon surgery. EPI-TEA group, TAP-TAP block group

No significant difference in maximal pain scores between groups, either on the day of surgery, on POD 1 or on POD 2, was found (Table 3). On the day of surgery, patients in TAP group declared higher pain scores at rest than those in the TEA group, but not statistically significantly. On PODs 1 and 2, the pain NRS score curves, measuring pain at rest, decreased more steeply in the TAP group than in the TEA group (Fig. 2A). The pain NRS score curves, measuring pain on movement, were equal in both groups on PODs 1 and 2 (Fig. 2B).

**Table 3:** Maximal pain (NRS 0–10)

	TAP *n* = 71	TEA *n* = 65	*p*
0 POD	4 (4.4)	4 (4.1)	0.4
1 POD	4 (3.6)	4 (4.1)	0.2
2 POD	2( 2.3)	2 (2.7)	0.3

Values are median (mean)

*NRS* numeric rate scale, 0-no pain, 10-worst pain, *POD* postoperative day

The mean of the patient satisfaction score (scale 0–3) in postoperative pain management at discharge was 2.8 (range 0–3) in both groups.

At 4 weeks postoperatively, median overall satisfaction scores (scale 0–3) in pain management were similar between groups (TEA 3, IQR 3–3, range 0–3 vs TAP 3, IQR 3–3, range 1–3: *p* = 0.11, data not shown). In agreement, questionnaires revealed no difference in daily pain sensation (pain NRS scores) or in pain-associated disability (sports, daily activities, social activity, mental wellbeing, sleeping) between groups.

The power of epidural analgesia was compared in pre-hoc-designed subgroups for right (*n* = 66) and left colectomies (*n* = 70). There was no difference in overall opioid consumption (cumulative mean opioid requirement) during the first 48 h postoperatively in either group between right and left colectomies (data not shown).

### Bowel function and mobilization

On the day of surgery, bowel function onset (gas or solid excrement) was registered for 10 patients (15.4%) in TEA group and 3 (4.2%) in TAP group (OR 4.2, 95% CI 1.1–15.8, *p* = 0.027); cumulatively on POD 1, 47 (72%) in the TEA and 45 (63%) in the TAP groups (OR 1.5, 95% CI 0.7–3.2, *p* = 0.2), and for POD 2, 63 (97%) in the TEA and 69 (97%) in the TAP groups (OR 0.9, 95% CI 0.1–14.9, *p* = 0.9).

On the day of surgery, 84.6% in the TEA group and 90% of patients in the TAP group (OR 1.6, 95% CI 0.6–4.5, *p* = 0.4) were mobilized, either in the recovery room or in the ward. On PODs 1 and 2, all (100%) were mobilized in the TEA group and all but one (99%) in the TAP group. Reasons for unsuccessful mobilization on the day of surgery were PONV, abdominal pain, weakness of lower limbs (in TEA group), confusion, and anal hemorrhagia.

There was no difference in mean fluid intake between the TEA and TAP groups on the day of surgery (2484 ml, range 1000–4300 ml vs 2274 ml, range 1000–3600 ml, respectively: *p* = 0.1).

### Complications and reoperations

The number of both severe (Clavien–Dindo 3–4) complications and re-operations was higher in the TAP than in the TEA group, although not statistically significantly. The rate of non-surgical complications did not differ between groups (Table 4).

**Table 4:** Postoperative complications

	TAP *n* = 71	EPI *n* = 65	*p*
Complications (Clavien–Dindo) (%)			0.5
1	2 (2.8)	3 (4.6)	
2	1 (1.4)	4 (6.2)	
3–4	7 (9.8)	3 (4.6)	
Complications (%)			
Ileus	2(2.8)	1(1.5)	
Anastomosis leakage	3(4.2)	1(1.5)	
Abdominal cavity abscess	1(1.4)	1(1.5)	
Anastomosis stricture	–	1(1.5)	
Haemorrhagia ex anus	1(1.4)	1(1.5)	
Anastomosis bleeding	1(1.4)	2(3.0)	
Other complications (%)			0.7
Pneumonia	1 (1.4)	1 (1.5)	
PONV	17 (23.9)	14 (21.5)	
Urine-retention	3 (4.2)	3 (4.6)	
other (AMI, FA, fever, diarrhea)	5 (7.0)	4 (6.2)	
Re-operation (%)	5 (7.0)	2 (3.1)	0.3
Re-admission in 30 days (%)	5 (7.0)	4 (6.2)	0.8

*PONV* postoperative nausea and vomiting, *AMI* acute myocardial infarction, *FA* atrial fibrillation

### LOS and readmissions

The median LOS was equal in both TAP and TEA groups (4 days, IQR 3–5, range 3–29 vs 4 days, IQR 4–5, range 2–18, respectively: *p* = 0.4). Similarly, rates of 30 days re-admissions were equal between groups (7% vs 6%, respectively, OR 0.9, 95% CI 0.2–3.4, *p* = 0.9) (Table 4).

## Discussion

One of the most important and influential items in all ERAS programs is effective pain management. TEA has been acknowledged as the gold standard for years for its benefits in decreasing bowel paralysis and stress response [18–20]. For elective colorectal surgery, this issue has been widely studied and discussed in diverging settings [10, 21, 22]. Our study is the first to compare purely ropivacaine-based TAP block and TEA in pain management after elective laparoscopic colon surgery in the setting of a randomized controlled trial (RCT).

According to the latest ERAS Society recommendations for colorectal surgery, TEA is recommended for open surgery but not as the first choice for minimally invasive surgery [23]. This is due to its invasive nature, potential adverse effects and complications, and risk of failure [24]. Pain management is better served by alternative methods, such as abdominal wall blocks. In 2016, a meta-analysis by Borzellino et al. showed LOS to be a day longer in the TEA group than with other blockades without other special advantages [25, 26]. Pirrera et al. compared TEA (ropivacaine and morphine) to TAP blockade in laparoscopic colon surgery and demonstrated less PONV and postoperative ileus in the TAP group [27]. This might partially relate to opioid-containing epidurals. In this study with purely ropivacaine-based TEA and TAP block, we observed faster onset of bowel function in the TEA group and no difference in PONV.

Both pain relief techniques, TEA and TAP block, have been shown to reduce surgical stress in postoperative pain management [28, 29]. This puts consideration of the method of postoperative analgesia into perspective since the emphasis is strongly on its safety. Utilizing the ERAS protocol has made postoperative recovery so prompt and effective that the benefits of TEA are likely to remain marginal. According to our results, the opioid-sparing effect of epidural analgesia is limited to the day of laparoscopic colon surgery. On the first POD, TAP block is not inferior to TEA and on the second POD, TAP was opioid-sparing in comparison to TEA, in this study. The question of whether any blockade is necessary after elective laparoscopic colorectal surgery was answered by Zhao et al., who compared postoperative TAP block with saline [30]. They reported decreased need for rescue opioids, faster bowel function onset, faster mobilization, and shorter LOS, with TAP block. With this background, our results support TAP block as a routine pain regimen in elective laparoscopic colon surgery and support the statement of the most recent ERAS guidelines in colorectal surgery restricting the use of TEA to specific well-reasoned situations.

This study’s strengths include the following: Study groups were identical in number, demographics, and perioperative characteristics; the same local anesthetic was used in both groups; perioperative care was carried out according to our updated ERAS protocol and the results should therefore be applicable in best current clinical practice.

Limitations include the following: for ethical and technical reasons, this study is not double blinded; not all patients received NSAIDs (on the other hand, all patients have been treated according to ERAS protocol, and therefore, this reflects real clinical situation); a continuing TEA infusion was compared with a single-shot TAP block (though this reflects again current clinical practice), and the results of this study can be applied for colorectal patients.

In conclusion, this is the first study to compare purely ropivacaine-based TAP block and TEA in pain management after elective laparoscopic colon surgery in the setting of an RCT and performed within an ERAS program. We report a significant reduction in opioid consumption and faster onset of bowel function on the day of surgery with TEA compared to TAP block. However, on POD 1, TAP block was not inferior to TEA in both opioid demand and bowel function. On POD 2, the opioid demand was significantly reduced with TAP block compared with TEA. Overall patient satisfaction with pain management was similar between groups and no blockade-related complications were detected.

## Supplementary Information

Below is the link to the electronic supplementary material.

Supplementary file1 (XLSX 41 KB)
